# The Dark Matter of Large Cereal Genomes: Long Tandem Repeats

**DOI:** 10.3390/ijms20102483

**Published:** 2019-05-20

**Authors:** Veronika Kapustová, Zuzana Tulpová, Helena Toegelová, Petr Novák, Jiří Macas, Miroslava Karafiátová, Eva Hřibová, Jaroslav Doležel, Hana Šimková

**Affiliations:** 1 Institute of Experimental Botany, Centre of the Region Haná for Biotechnological and Agricultural Research, Šlechtitelů 31, CZ-78371 Olomouc, Czech Republic; kapustova@ueb.cas.cz (V.K.); tulpova@ueb.cas.cz (Z.T.); toegelova@ueb.cas.cz (H.T.); karafiatova@ueb.cas.cz (M.K.); hribova@ueb.cas.cz (E.H.); dolezel@ueb.cas.cz (J.D.); 2 Biology Centre, Czech Academy of Sciences, Institute of Plant Molecular Biology, Branišovská 31, CZ-37005 České Budějovice, Czech Republic; petr@umbr.cas.cz (P.N.); macas@umbr.cas.cz (J.M.)

**Keywords:** genome assembly, bread wheat, barley, optical mapping, BAC, ribosomal DNA

## Abstract

Reference genomes of important cereals, including barley, emmer wheat and bread wheat, were released recently. Their comparison with genome size estimates obtained by flow cytometry indicated that the assemblies represent not more than 88–98% of the complete genome. This work is aimed at identifying the missing parts in two cereal genomes and proposing techniques to make the assemblies more complete. We focused on tandemly organised repetitive sequences, known to be underrepresented in genome assemblies generated from short-read sequence data. Our study found arrays of three tandem repeats with unit sizes of 1242 to 2726 bp present in the bread wheat reference genome generated from short reads. However, this and another wheat genome assembly employing long PacBio reads failed in integrating correctly the 2726-bp repeat in the pseudomolecule context. This suggests that tandem repeats of this size, frequently incorporated in unassigned scaffolds, may contribute to shrinking of pseudomolecules without reducing size of the entire assembly. We demonstrate how this missing information may be added to the pseudomolecules with the aid of nanopore sequencing of individual BAC clones and optical mapping. Using the latter technique, we identified and localised a 470-kb long array of 45S ribosomal DNA absent from the reference genome of barley.

## 1. Introduction

Small grain cereals, such as bread wheat, durum wheat, barley and rye are crucial crops for the European population and most of them are grown worldwide. Despite their socio-economic importance, reference genomes of these cereals were only recently generated [1,2,3,4,5], which can be attributed to their high genome complexities, ranging from ~5 Gb for barley to ~16 Gb for bread wheat [6], and enormous proportion of repetitive DNA (85–90%). These assemblies are characterized by high contiguity and low proportion of internal gaps. However, a comparison of assembly lengths with genome-size estimates obtained by flow cytometry indicated that the reference genomes of barley, wild emmer wheat, bread wheat and rye represented no more than 98%, 88%, 90% and 90% of the estimated genome sizes, respectively [6]. This gives rise to an obvious question as to what the missing part of the reference genomes is, and stimulates efforts towards complementing it.

Low-copy genome regions are known to be a relatively easy target for genome assemblers and were found well represented even in early cereal genome sequences that were based on low-coverage Roche/454 data [7,8,9] or Illumina pair-end reads only [10]. On the contrary, large regions of repeats are known to pose a challenge and result in gaps, mis-assemblies and collapsed tandem repeats in a majority of genome sequences [11]. Dispersed repeats, represented by various types of transposable elements, have been largely resolved in the recent assemblies thanks to the combination of pair-end and mate-pair Illumina reads and sophisticated assembling algorithms [1,3] or implementation of long-read PacBio data [12].

On the other hand, tandem repeats organized as arrays of multiple units (microsatellites, macrosatellites and centromeric satellite repeats) tend to collapse in assemblies into fewer copies. Consequently, they are under-represented in reference genomes and pose a significant source of gaps and assembling errors in de novo assemblies, including that of humans [11]. The repeat-associated gaps are abundant in heterochromatic regions, making it impossible to completely assemble these genome parts. This usually results in genome assemblies missing a majority of (peri)centromeric regions and secondary constrictions [11,13]. To resolve the copy numbers, it is essential to use reads longer than the total array length. To some extent, this can be sorted out by using long-read DNA sequencing technologies, such as PacBio or nanopore sequencing, which produce reads of tens and up to hundreds kilobases, respectively. Nevertheless, only short arrays of simpler repeats, such as 5S rRNA multigene loci spanning over several kilobases, can be tackled by these approaches [14]. Arrays spanning over hundreds to thousands kilobases and consisting of units that are several kilobases long, such as loci coding for 45S rRNA, cannot be resolved by any of the current sequencing technologies. 

This shortage can be compensated for by other approaches that facilitate identification, positioning and characterization of long arrays of tandem repeats, such as in situ hybridisation, application of dedicated bioinformatics tools, and optical mapping. The initial methods to investigating distribution of tandem repeats in cereal genomes included in situ hybridisation (ISH) [15,16,17] and fluorescence in situ hybridisation (FISH) [18,19]. Although the cytogenetic techniques provided first insights into the genome evolution [20] and facilitated chromosome identification and construction of molecular karyotypes [16,19,21], they did not provide information at the DNA sequence level. This could be obtained by application of dedicated bioinformatics tools, such as RepeatExplorer [22,23]. This computational pipeline utilizes a graph-based sequence clustering algorithm to de-novo assemble tandem repeats from raw next-generation sequencing data, without the need of a reference database of known elements. It has been used to identify and characterize repetitive elements in several complex plant genomes, including that of rye [24]. 

Optical mapping in nanochannel arrays, also known as Bionano genome (BNG) mapping, is a high-throughput long-read technology that generates genome maps of a short sequence motif—the recognition site of an enzyme used for labelling [25]. It has been used to support and validate physical-map and genome assemblies of several complex cereal genomes [2,3,26,27,28]. The ability of optical mapping to size gaps, cover some types of tandem DNA repeats [26] and identify misassemblies due to collapsed duplicated sequences [29] makes this technology a promising tool for identifying and characterizing missing parts of genome assemblies. 

In this study, we identified three new tandem repeats specific for the short arm of wheat chromosome 7D (7DS) and interrogated their representation in recently published bread wheat assemblies, including (i) Triticum 3.1 [12], which combines short Illumina and long PacBio reads, (ii) IWGSC RefSeq v1.0 [3], which is based on short reads only, and (iii) Illumina assemblies of physical map-ordered 7DS-specific BAC clones [30]. While these assemblies comprised of arrays of all three repeats, they failed in unravelling organization of a repeat with unit size of 2726 bp, whose genome arrangement could only be resolved after adding information from nanopore sequencing of two BAC clones bearing arrays of this repeat and by using an optical map (OM) of the wheat 7DS arm [26] as a reference. Besides, we employed an OM of barley cv. Morex [31] to investigate a minor 45S rDNA locus in barley chromosome 1H, which was identified by in situ hybridisation in various barley cultivars [32,33] and underrepresented in the ’Morex’ BAC-by-BAC genome assembly [2]. Thus, we demonstrated that optical mapping is a suitable tool to identify the missing parts of the assemblies, and, in some cases, can reveal overall organization of the repeat array. Targeted long-read nanopore sequencing was confirmed as a promising approach to complementing the missing sequences to the genome assemblies.

## 2. Results and Discussion

### 2.1. Chromosome-Specific Tandem Repeats in Wheat 

Using RepeatExplorer pipeline to cluster Illumina raw data from flow-sorted wheat chromosome arm 7DS, we identified four tandem repeats with monomer lengths ranging from 1167 bp to 2726 bp (Table 1, File S1). 

**Figure 1:**
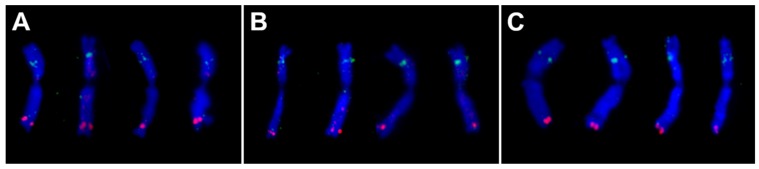
FISH on metaphase 7D chromosomes of bread wheat cv. Chinese Spring with probes for GAA microsatellite (green) and three tandem repeats (red). (**A**) TaeCsTr163, (**B**) TaeCsTr230 and (**C**) TaeCsTr99 repeats localized in subtelomeric region of the 7DS chromosome arm. 7D chromosomes were identified based on the GAA hybridization signal on the 7DL arm. The chromosomes were counterstained by DAPI (blue).

Cytogenetic mapping revealed that three of the repeats, TaeCsTr163, TaeCsTr230 and TaeCsTr99, provided unique FISH signals specific for subtelomeric region of wheat 7DS chromosome arm (Figure 1; Figure S1). A probe derived from the TaeCsTr111 sequence provided dispersed hybridization signals on multiple chromosomes, predominantly in pericentromeric and subtelomeric regions (Figure S1). The clustered hybridization signals obtained with three probes are supportive of the tandem organisation of these repeats. Application of RepeatExplorer on raw data obtained from flow-sorted chromosomes thus proved a suitable approach to identify chromosome-specific tandem repeats. The tool worked efficiently, despite using DNA amplified by multiple displacement amplification, which is known to introduce a quantitative amplification bias [34]. 

To assess the representation of the tandem repeats in the recently published wheat reference genome, we performed blastn search on IWGSC RefSeq v1.0 assembly [3]. Out of the three repeats assigned specifically to the 7DS arm, only two, TaeCsTr163 and TaeCsTr230, could be reliably identified in the IWGSC RefSeqv1.0 assembly of the 7D chromosome. Using blastn search, we found a cluster of 39 complete and several incomplete units of the TaeCsTr163 repeat, partially tandemly organised, that spanned over 260 kb in the interval of 49.08–49.34 Mb of the 7D pseudomolecule. The TaeCsTr230 repeat was identified as an array of ten complete and three incomplete units located in the interval of 33.254–33.27 Mb of the 7D pseudomolecule (Figure S2). Considering the entire 7DS arm length of 338 Mb [3], the positions of both repeats in the assembly are in agreement with their cytogenetic locations. On the contrary, we failed to find a significant blastn hit in the 7D pseudomolecule of RefSeq v1.0 for the repeat TaeCsTr99 that provided the strongest FISH signal on mitotic chromosomes.

**Figure 2:**
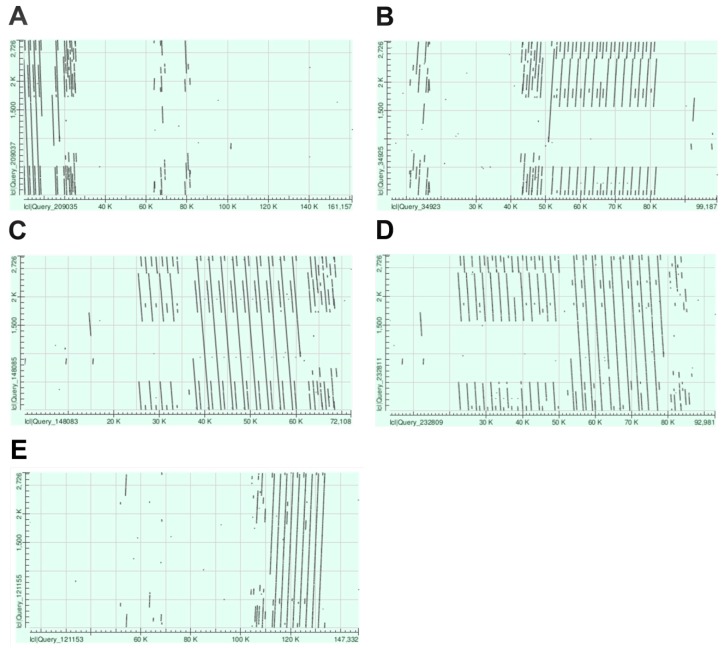
Dot plots showing position and arrangement of TaeCsTr99 repeat in (**A**) ChrUn8536, (**B**) interval 9.06 Mb to 9.16 Mb of 7D pseudomolecule of Triticum 3.1 assembly [12] (**C**) 72-kb Illumina contig of BAC clone 28N04, (**D**) 99.8-kb nanopore read 51ef9015 of BAC clone 28N04, (**E**) 148-kb nanopore read f24cdcf5 of BAC clone 104G18.

**Figure 3:**
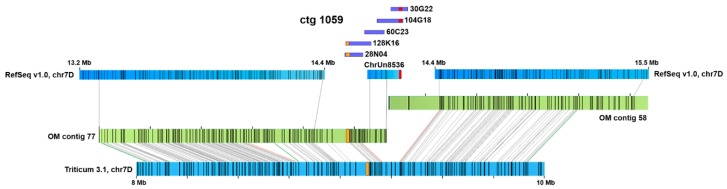
Organization of the TaeCsTr99 region. Positions of two TaeCsTr99 arrays (highlighted in orange and red) and an overall arrangement of the region were obtained by aligning ChrUn8536, IWGSC RefSeq v1.0 [3] and Triticum 3.1 [12] 7D pseudomolecules (blue bars) and BAC clones of 7DS physical-map contig 1059 [30] (violet bars) to 7DS optical map [26] (green bars). Numbers at the 7D pseudomolecules indicate assembly coordinates.

Additional search in the unassigned scaffolds (ChrUn) revealed 12 of them containing TaeCsTr99 units (Figure 2A, Figure S3). Scaffold lengths varied from 3 kb to 163 kb, and they comprised from one to eleven complete TaeCsTr99 units. Altogether, we identified in unassigned scaffolds 36 complete tandemly organised units totalling 98 kb of length, accompanied by several incomplete units and unit fragments. Based on a high sequence homology (Figure S3), some of the scaffolds could be overlapping. Out of the twelve scaffolds, only ChrUn8536 carried an 82.3-kb non-repetitive segment (Figure S4A), which enabled its positioning in the context of the 7D pseudomolecule utilizing the OM of the 7DS chromosome arm. OM contig 77 placed the ChrUn8536 to position 14.4 Mb in the 7D pseudomolecule and revealed an additional 272 kb gap distal of the ChrUn8536 (Figure S5A, Figure 3). The identified position is consistent with the (sub)telomeric location of the repeat indicated by FISH (Figure 1C).

In order to span the entire TaeCsTr99 array, we employed short-read sequence assemblies of physical-map ordered 7DS BAC clones [30]. TaeCsTr99 was identified in four BAC clones belonging to 7DS physical-map contig 1059. Clones 104G18 and 30G22 overlap and show sequence homology with ChrUn8536 (Figure 3). Illumina assemblies of these BAC clones were rather fragmented and did not allow reconstructing the entire TaeCsTr99 array. Surprisingly, an array of the TaeCsTr99 repeat was also found in overlapping BAC clones 28N04 and 128K16. The latter could be aligned to the OM 77 (Figure S5B), but it was separated from the TaeCsTr99 array identified in clones 104G18 and 30G22 by a 225-kb non-repetitive segment (Figure 3, Figures S4 and S5). This suggested the presence of two separate TaeCsTr99 arrays in a close proximity, which we termed distal (covered by clones 128K16 and 28N04) and proximal (covered by ChrUn8536, 104G18 and 30G22), respectively. The size of the distal array was deduced from the assembly of BAC clone 28N04, which appeared to comprise the entire array in one scaffold, and was estimated to be ~44 kb. This array was composed of nine complete units, four units comprising a 1056-bp deletion and an additional cluster of repeat fragments spanning over ~6 kb (Figure 2C). The size of the proximal array in ChrUn8536 was 26 kb and this, likely incomplete array sequence comprised of three complete and three partial units and a ~6-kb cluster of repeat fragments (Figure 2A). Apparently, the organization of the repeat array in BAC clones 28N04 and 128K16 differed from that in ChrUn8536 (Figure 2, Figure S4A), which supported our hypothesis of two spatially separated arrays of TaeCsTr99 repeat in 7DS. 

We hypothesized that the difficulties in assembling and incorporating of the TaeCsTr99 arrays into the pseudomolecule could be overcome by employing longer reads, such as those generated by SMRT sequencing (PacBio technology). To verify this, we explored bread wheat Triticum 3.1 assembly [12] that combines short-read Illumina and long-read PacBio data to search the 7D pseudomolecule for the TaeCsTr99 sequence. The blastn search revealed in the position 9.11 - 9.14 Mb a ~30-kb array composed of 14 incomplete TaeCsTr99 units, 13 of which carried the 1056-bp deletion observed in BAC clone 28N04 (Figure 2). We also observed the ~6-kb cluster of repeat fragments located distal of the array. Alignment of this region to 7DS OM placed the array to position ~1.2 Mb in OM contig 77 (Figure S5C), which corresponded to the position of the distal array identified in the short-read assemblies (Figure 3). On the contrary, we did not find any evidence of the proximal array in the Triticum 3.1 assembly. 

To resolve the discrepancies in the location and organization of the TaeCsTr99 arrays identified in various assemblies, we made use of the long-read platform of Oxford Nanopore Technologies (ONT) and generated nanopore reads from BAC clones 28N04 and 104G18, which cover the distal and proximal array, respectively. For each of the clones, we obtained two reads that spanned over the entire insert and showed a consistent array structure. ONT read 51ef9015 (File S2) of 99,802 bp covering clone 28N04 confirmed the complex structure of the distal array composed of two sub-arrays with differently organised units (Figure 2D, Figure S4B). The distally located sub-array, approximately 27 kb in size, comprised of 10 complete units of the TaeCsTr99. The proximal sub-array was of similar length and consisted of 12 incomplete units, bearing a distinct deletion between 507 and 1563 bp of the TaeCsTr99 sequence. The ONT read also confirmed the presence of the adjacent ~6-kb cluster consisting of TaeCsTr99 fragments, which was less obvious here than in the Illumina BAC assembly due to the inherent inaccuracy of the nanopore technology (Figure 2C,D). Except for the variation in the number of units, the overall structure of the distal array looked highly similar in the BAC Illumina assembly and the ONT read. On the contrary, the corresponding array in whole-genome Triticum 3.1 assembly differed by the absence of the full-length units (Figure 2B). The proximal array was covered by a 148,009 bp full-length read f24cdcf5 of clone 104G18 (File S2), comprising the entire array that spanned over ~30kb and had a simple structure, including eight complete and one incomplete unit and the cluster of repeat fragments (Figure 2E). The total number of TaeCsTr99 units in the ONT reads covering the distal and the proximal array (18) was smaller than that identified in unassigned scaffolds (36). This could be due to non-recognized overlaps between the scaffolds, which may have resulted in overestimating the number of the repeats. Alternatively, we cannot exclude the presence of additional TaeCsTr99 array(s), missing both in the pseudomolecules and in the 7DS BAC assemblies that might be located in proximity of the confirmed ones. 

The data obtained in our study suggest that tandemly organised repeats with unit size of 1–3 kb are not the major contributor to the missing part of the wheat IWGSC RefSeq v1.0 assembly as three of such repeats were well represented in the wheat reference genome obtained from short read data. Nevertheless, a more detailed analysis of a repeat with unit size of 2726 bp revealed that it was completely missing from the 7D pseudomolecule and was found in unassigned scaffolds (ChrUn) of the RefSeq v1.0 only. Thus, we concluded that this type of repeats may cause shrinking of pseudomolecules without impacting size of the entire assembly. Our results are in line with a finding that 27% centromeric sequences, identified by association with a centromere-specific histone H3 variant and highly enriched in centromere-specific repeats, were found in ChrUn of the RefSeq v1.0 [3]. This indicates that advanced assemblers can to some extent assemble shorter arrays of tandemly organised repeats but integration of these arrays in the pseudomolecule context may still pose a substantial challenge. 

The TaeCsTr99 repeat was also found underrepresented and likely misassembled in the 7D pseudomolecule of Triticum v3.1, generated from both Illumina and PacBio reads. Moreover, both tested wheat whole-genome assemblies failed in discriminating two similar arrays located 225 kb apart, which could only be resolved after nanopore sequencing of BAC clones. This approach was successful not only because it employed a technology that provides reads exceeding the length of the whole array, but also because it leveraged the separation of the two arrays into the individually sequenced BAC clones. Interestingly, the identification of relatively long arrays of tandem repeats in BAC clones contradicts the finding of [11] that the tandem repeats are underrepresented in BAC libraries because of their toxicity for bacteria. The organisation of the whole tandem arranged region was resolved thanks to the application of the OM of the 7DS arm, which provided a reference for alignment of various sequences and revealed existing gaps and misassemblies. Nevertheless, the full potential of this genomic resource could not be exploited because none of the repeats analysed comprised a *Bsp*QI site (GCTCTTC) labelled in the 7DS OM. Consequently, the repeat arrays could not be recognised in the map through a specific labelling pattern, but appeared as longer regions devoid of labels. This shortage of the method might be overcome by the application of a new approach based on CRISPR-mediated labelling of specific sequences in the context of the optical map [35], which may facilitate straightforward mapping and quantifying of any repeat of interest. 

### 2.2. Minor 45S rDNA Locus in Barley Chromosome 1H

**Figure 4:**
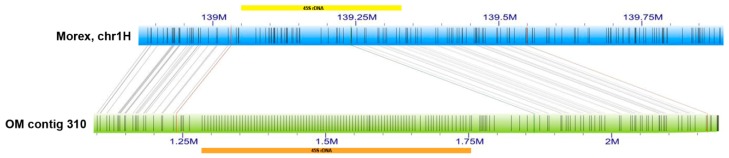
Positioning of the 45S rDNA locus in barley chromosome1H. Alignment of barley ‘Morex’ 1H pseudomolecule [2] (blue bar) to ‘Morex’ OM contig 310 (green bar) revealed a tandemly organized repeat with ~5 kb label spacing (highlighted by orange bar) missing in the pseudomolecule. Co-localisation with a cluster of 45S rDNA fragments in the sequence assembly (highlighted by yellow bar) indicates that the array represents the 1H rDNA locus.

Our second target was a minor 45S ribosomal DNA locus in barley chromosome 1H, identified by in situ hybridisation in various barley cultivars [32,33]. To access this locus, we first reconstructed the 1H-specific rDNA unit from 1H-specific paired-end Illumina reads (File S3). The unit sequence with the length of 8407 bp was then used to search 1H pseudomolecule of the barley ‘Morex’ reference genome [2]. Fragments of the unit were found between 139.05 Mb and 139.33 Mb of the 1H pseudomolecule, which fits well with the cytogenetic location of the rDNA locus at ~60% of the short arm of 1H [32], but we did not identify a regular rDNA array at this locus. To investigate completeness of the sequence in this region, we aligned it to available OM of barley cv. Morex [2], which identified OM contig 310 spanning over the region (Figure 4). Central part of the contig 310 did not align to the pseudomolecule and showed a regular labelling pattern with label spacing of approximately 5 kb. We compared it with the label pattern predicted for tandemly organised rDNA units. The reconstructed rDNA unit sequence comprised of three *Bsp*QI sites (File S3), but two of them were located just 1133 bp apart, which is too close for them to be discriminated in optical maps generated on the Bionano Genomics Irys platform. Thus, the labels associated with the *Bsp*QI sites were predicted to generate a composed pattern alternating ~3.5- and ~4.9-kb units (Figure S6A). This roughly corresponded to the pattern seen in the OM, with a discrepancy relating to the predicted ~3.5 kb “restriction fragment”, which was not apparent in the optical map. This fragment covers the intergenic spacer (IGS) that comprises of two types of shorter tandem repeats with 78- and 135-bp unit length, respectively. It is likely that these repeats are collapsed in our consensus sequence and their real number is larger, extending the proposed IGS size by as much as 1.5 kb. This hypothesis was supported by the analysis of several partial rDNA units found in the 1H pseudomolecule, which were showing for both spacer repeats a higher number than included in our rDNA consensus sequence. Thus, we suggest that the complete size of the 1H rDNA unit is ~9.9 kb, which is supported by findings of [36] who identified in barley ribosomal DNA units of two sizes, 9.9 kb and 9 kb. Co-localisation of the blastn hits for 45S rDNA with the array in the optical map and the reported cytogenetic position lead us to the conclusion that the ~470-kb long array comprising ~47 putative rDNA units represents the minor 1H rDNA locus detected by in situ hybridisation. Our copy number estimate is close to that of [32] who quantified rRNA genes in the 1H chromosomes by in situ hybridization and proposed 50-100 copies in this locus. The slight discrepancy could be due to using a different barley cultivar (Morex vs. Sultan).

Using the optical map, we identified a 470-kb segment that is absent from the Morex 1H pseudomolecule. We also performed blastn search for the consensus 45S rDNA sequence in chromosomes 5H and 6H, which are known to harbour major barley rDNA loci comprising thousands of genes [32,36], and in unassigned scaffolds of the ‘Morex’ assembly [2]. The search failed in revealing rDNA arrays in any of the datasets and identified fragments of the rDNA units only. This indicates that the missing rDNA loci contribute significantly to the dark matter of the cereal genomes. 

## 3. Materials and Methods

### 3.1. De Novo Identification of Wheat Tandem Repeats

In order to identify new tandem repeats specific for the short arm of wheat chromosome 7D (7DS), we randomly selected 3.6 million reads obtained by Illumina sequencing multiple-displacement-amplified (MDA) DNA of flow-sorted 7DS [37]. Raw reads were examined and filtered by quality using FastQC and Trimmomatic tool. Repeat identification was performed employing similarity-based clustering of paired-end (2 × 100 nt) Illumina reads using local installation of the RepeatExplorer pipeline [23]. The pipeline employs graph representation of read similarities to find clusters of frequently overlapping reads corresponding to various repetitive elements or their parts. Putative tandem repeats were identified based on circular topology of their graphs [22] and tandem structure of contigs assembled from the reads within individual clusters. Sequences of the assembled contigs were then used to design PCR primers to verify the presence of corresponding sequences in the wheat genome (Table S1). In addition, the amplified fragments were cloned using the TOPO-TA Cloning Kit for Sequencing (Invitrogen, Carlsbad, CA, USA), selected clones were verified by sequencing and used as probes for in situ hybridisation experiments.

### 3.2. In Situ Hybridisation

For the in situ hybridisation experiment, we employed seeds of *Triticum aestivum* L., cv. Chinese Spring, kindly provided by Dr. Pierre Sourdille (INRA, Clermont-Ferrand, France). Seed germination, cell cycle synchronisation, metaphase accumulation and squash preparations were performed from wheat root tip meristems according to [38] with minor modifications. Metaphase accumulation was done by incubating root tips in 2.5 µM amiprophos-methyl for 2h in the dark at 25 °C. Inserts of clones bearing particular repeats were amplified using M13 primers and the PCR products were labeled by biotin using BioNick^™^ Labeling System (Invitrogen, Carlsbad, CA, USA). GAA microsatellite, used for identification of wheat chromosomes, was labelled by digoxigenin. Biotin- and digoxigenin-labeled probes were detected using streptavidin-Cy3 (Invitrogen, Carlsbad, CA, USA) and anti-digoxigenin-fluorescein (Roche, Basel, Switzerland), respectively. Chromosomes were counterstained with 4′,6′-diamidino-2-phenylindole (DAPI) and the preparations were imaged using Axio Imager Z.2 Zeiss microscope (Zeiss, Oberkochen, Germany) equipped with a CCD camera. 

### 3.3. Reconstruction of Barley 1H rDNA Unit

RepeatExplorer pipeline was used to perform reconstruction of 45S rDNA sequence of barley. To do this, whole-genome paired-end (2 × 100 nt) Illumina reads of barley (*Hordeum vulgare*) cv. Morex (SRR490932) were downloaded from the SRA database, trimmed to quality and used for graph-based clustering. The resulting barley consensus 45S rDNA sequence was then used as a guide for reconstruction of a barley 1H chromosome-specific 45S rDNA sequence. This was done using online version of RepeatExplorer pipeline on the Galaxy platform and applying paired-end (2 × 100 nt) Illumina reads from flow-sorted 1H chromosome [39] of *H. vulgare* cv. Morex (SRR490144). The graph-based clustering resulted in five clusters homologous to the barley consensus 45S rDNA. The 1H-specific rDNA unit was then assembled manually utilizing the barley consensus 45S rDNA as a reference. 

### 3.4. Application of Optical Maps

To validate sequences and analyse repeats in wheat 7DS and barley 1H chromosome, we employed available optical (BNG) maps constructed from 7DS chromosome arm of wheat cv. Chinese Spring [26] and the whole genome of barley cv. Morex [31], respectively. Both maps were assembled from single molecule data obtained after labelling molecules at Nt.*Bsp*QI nicking sites (motif GCTCTTC). Comparison of the optical maps with sequences was carried out using the IrysView 2.5.1 software package (Bionano Genomics, San Diego, CA, USA). For the alignment, cmap files were generated from fasta files of particular sequences. Query-to-anchor comparison was done with default parameters and *P*-value threshold of 1e^−10^. 

### 3.5. Nanopore Sequencing

To resolve organization of the TaeCsTr99 repeats in the wheat genome, nanopore sequencing was conducted on 7DS BAC clones TaaCsp7DS028N04 (28N04) and TaaCsp7DS104G18 (104G18) from the ‘Chinese Spring’ 7DS arm-specific BAC library [40]. BAC DNA was extracted using alkaline lysis method followed by phenol-chloroform extraction and ethanol precipitation. Finally, the DNA was purified by incubating with 1:1 AMPure XP beads (Beckman Coulter, Miami, FL, USA) for 5 min and eluted into 30 µl 10 mM Tris, pH 8.5. Barcoded sequencing libraries were prepared from 700 ng DNA per BAC clone using Rapid Barcoding Sequencing Kit (SQK-RBK004; Oxford Nanopore Technologies, Oxford, UK) and sequenced together with additional ten clones on the MinION platform (Oxford Nanopore Technologies, Oxford, UK). Raw data were basecalled using Poretools 0.6.0 (https://github.com/arq5x/poretools, accessed on: 30 May 2019), demultiplexed using Porechop 0.2.3 (https://github.com/rrwick/Porechop, accessed on: 30 May 2019) and size-filtered >10 kb, which yielded 315 reads ranging from 10,003 to 101,160 bp, and 62 reads ranging from 10,082 to 149, 812 bp for the clone 28N04 and 104G18, respectively. Selected reads of 99,802 bp and 148,009 bp for 28N04 and 104G18, respectively, spanned the entire lengths of the respective clones. 

## 4. Conclusions

Our study on tandem organised DNA repeats with unit sizes of 1.2–2.7 kb suggested that such repeats might be present in genome assemblies of large cereal genomes even if generated from short-read data. Nevertheless, they are typically comprised in short sequence contigs or scaffolds and thus may be difficult to incorporate into the pseudomolecules. We demonstrated that tandem repeats could be identified by a dedicated bioinformatics tool—RepeatExplorer—on a chromosome-specific basis and that nanopore sequencing of BAC clones provided a reliable approach to analysing organization of particular repeat arrays. We showed that an optical map might be useful for anchoring unassigned repeat-bearing scaffolds and for validating sequence assemblies in the problematic regions. The potential of the method was confirmed in our attempt to localise and characterise a minor 45S ribosomal DNA locus, which is missing in the reference genome of barley. Since BAC resources and optical maps are available for many plant species including major crops, the approaches presented in our study are widely applicable.

## Figures and Tables

**Table 1:** Monomer sizes and distribution of identified tandem repeats.

Tandem Repeat	Monomer Size	Distribution
TaeCsTr163	1390 bp	7D subtelomere
TaeCsTr230	1242 bp	7D subtelomere
TaeCsTr99	2726 bp	7D subtelomere
TaeCsTr111	1167 bp	All chromosomes Dispersed

## Supplementary Materials

Supplementary materials can be found at https://www.mdpi.com/1422-0067/20/10/2483/s1.

## Abbreviations

7DS	Short arm of wheat chromosome 7D
BAC	Bacterial Artificial Chromosome
BNG mapping	Bionano genome mapping
CRISPR	Clustered Regularly Interspaced Short Palindromic Repeats
DAPI	4,6-diamidino-2-phenylindole
FISH	Fluorescence in situ hybridisation
IGS	Intergenic spacer
ISH	In situ hybridisation
IWGSC	International Wheat Genome Sequencing Consortium
MDA	Multiple Displacement Amplification
OM	Optical map
ONT	Oxford Nanopore Technologies
PacBio	Pacific Biosciences
rDNA	Ribosomal DNA
SMRT	Single-molecule real-time
SRA	Sequence read archive
