# Supplementary material for: The Dark Matter of Large Cereal Genomes: Long Tandem Repeats
Source: Int J Mol Sci. 2019 May 20;20(10):2483. doi: 10.3390/ijms20102483 (PMC6567227; doi:10.3390/ijms20102483)
Supplement: Supplementary file 1 [file ijms-20-02483-s001.zip › suppl.pdf]

Supplementary Materials

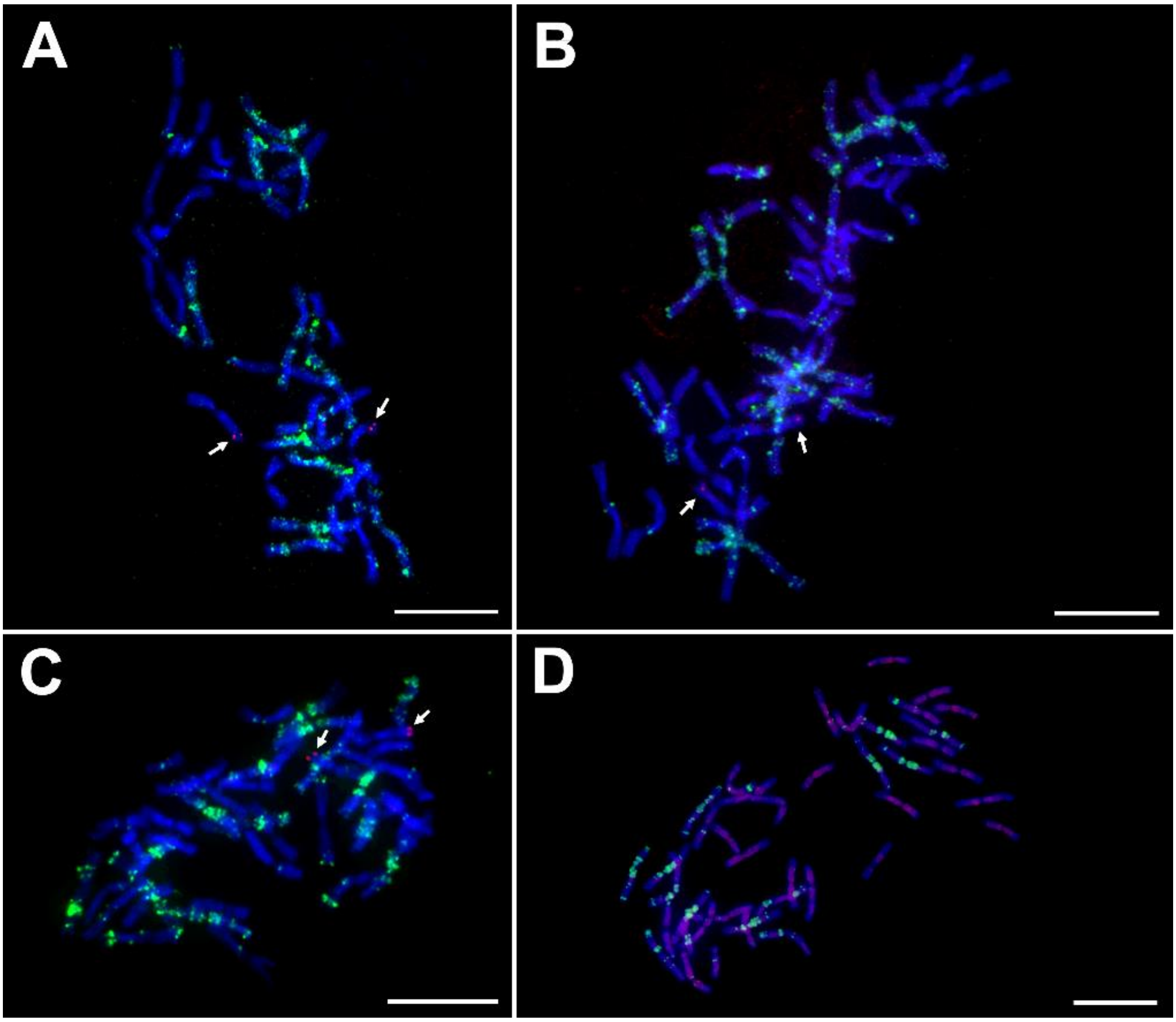

Figure S1. FISH on metaphase chromosomes of bread wheat cv. Chinese Spring with probes for GAA microsatellite (green) and particular tandem repeats (red). TaeCsTr163 (A), TaeCsTr230 (B) and TaeCsTr99 (C) repeats provided distinct signals in subtelomeric region of the 7DS. The TaeCsTr111 repeat (D) provided non-specific signals in pericentromeric and subtelomeric regions of multiple chromosomes. 7D chromosomes were identified based on the GAA signal on the 7DL arm. Signals of the tandem repeats are indicated by arrows. Scale bars represent 10 µm.

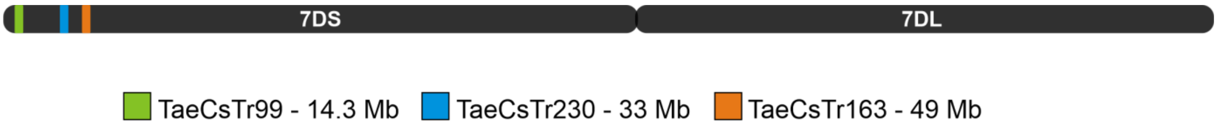

Figure S2. Positions of clusters of tandem repeats TaeCsTr99, TaeCsTr230 and TaeCsTr163 in the RefSeq v1.0 7D pseudomolecule [3]. Ten and 39 complete units were found for TaeCsTr230 and TaeCsTr163, respectively. No complete unit was identified in the pseudomolecule for the TaeCsTr99.

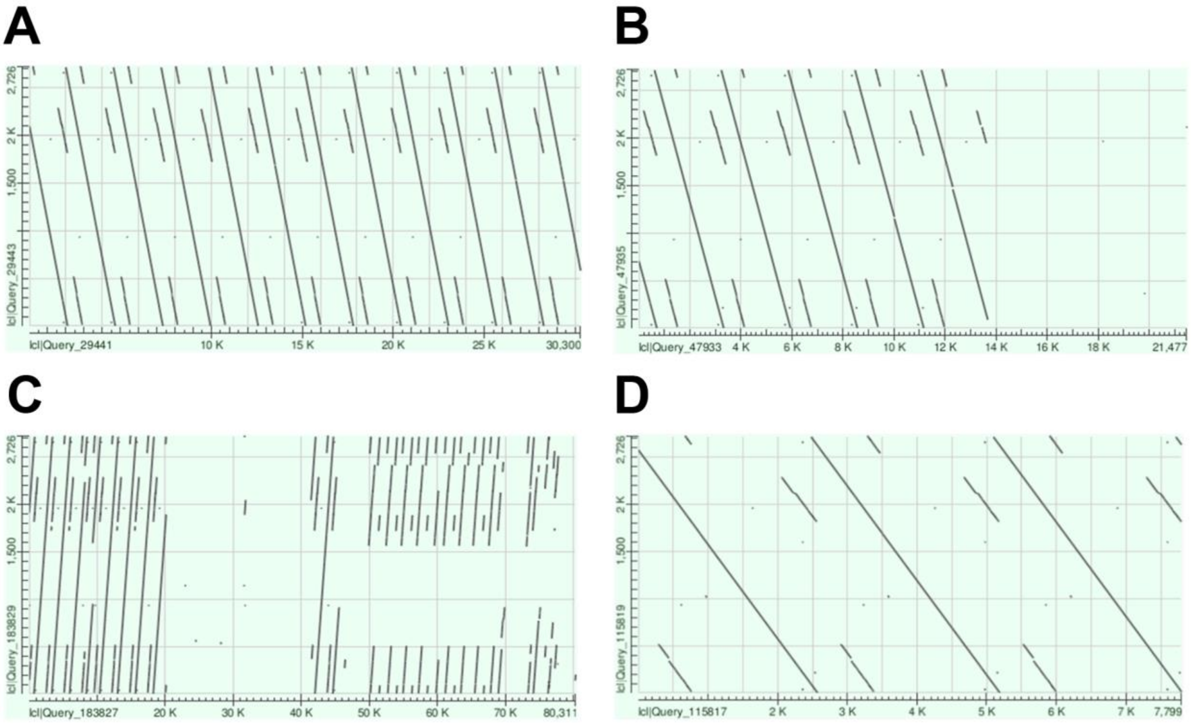

Figure S3. Organisation of the TaeCsTr99 repeat in selected wheat RefSeq v1.0 [3] unassigned scaffolds (ChrUn). Dot plots were obtained after alignment of the TaeCsTr99 repeat sequence with sequences of unassigned scaffolds. (**A**) ChrUn35180, (**B**) ChrUn39073, (**C**) ChrUn24017, (**D**) ChrUn18007.

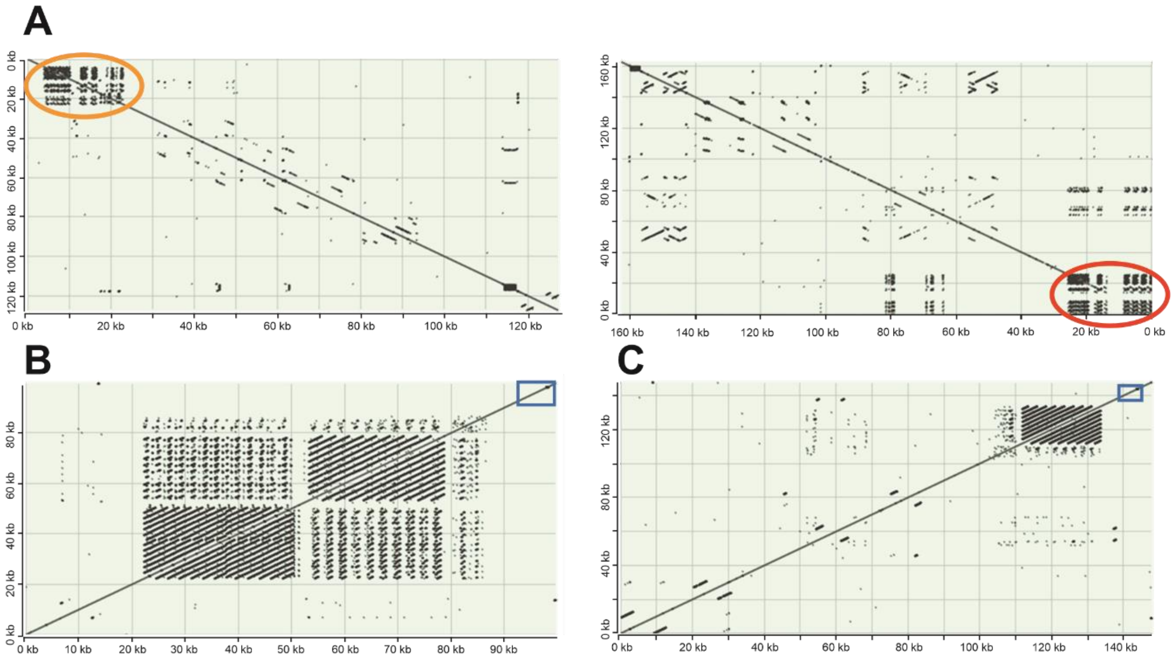

Figure S4. Organisation of the TaeCsTr99 repeat region bearing a distal and a proximal sub-array. Dot plots were obtained after self-alignment of the sequences. (**A**) Illumina contig of BAC clone 128K16 (left) and RefSeq v1.0 unassigned scaffold ChrUn8536 (right). The distal and the proximal array are highlighted orange and red, respectively. (**B**) ONT read 51ef9015 of BAC clone 28N04 and (**C**) ONT read f24cdcf5 of BAC clone 104G18. BAC vector sequence is indicated by the blue box.

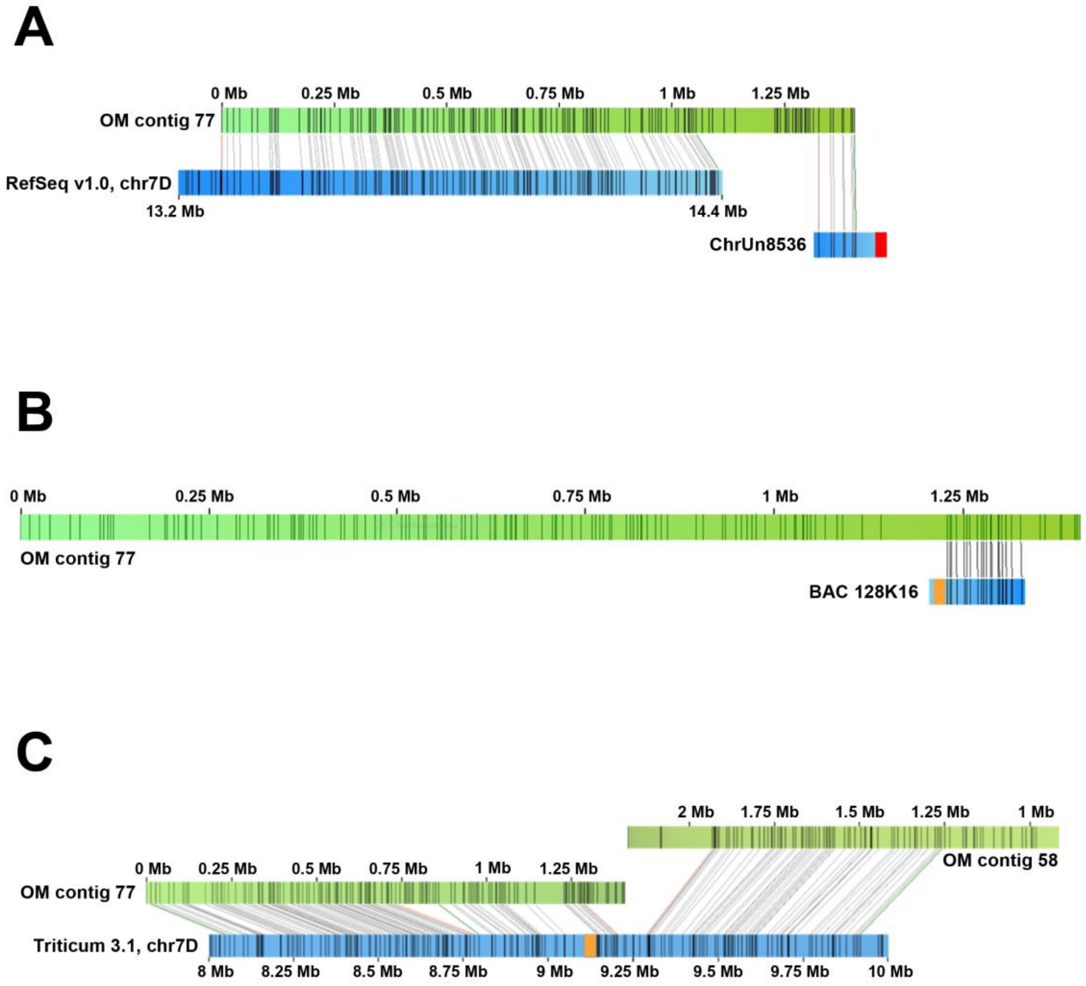

Figure S5. Alignment of TaeCsTr99-bearing sequences to 7DS optical map. (**A**) Alignment of scaffold ChrUn8536 to IWGSC RefSeq v1.0 7D pseudomolecule [3] through OM contig 77. (**B**) Alignment of BAC clone 128K16 to OM contig 77. (**C**) Alignment of OM contigs 77 and 58 to Triticum 3.1 7D pseudomolecule [12]. Sequences and optical maps are shown as blue and green bars, respectively. Positions of TaeCsTr99 arrays in the sequences are highlighted red and orange, respectively. The numbers below the pseudomolecules indicate assembly coordinates.

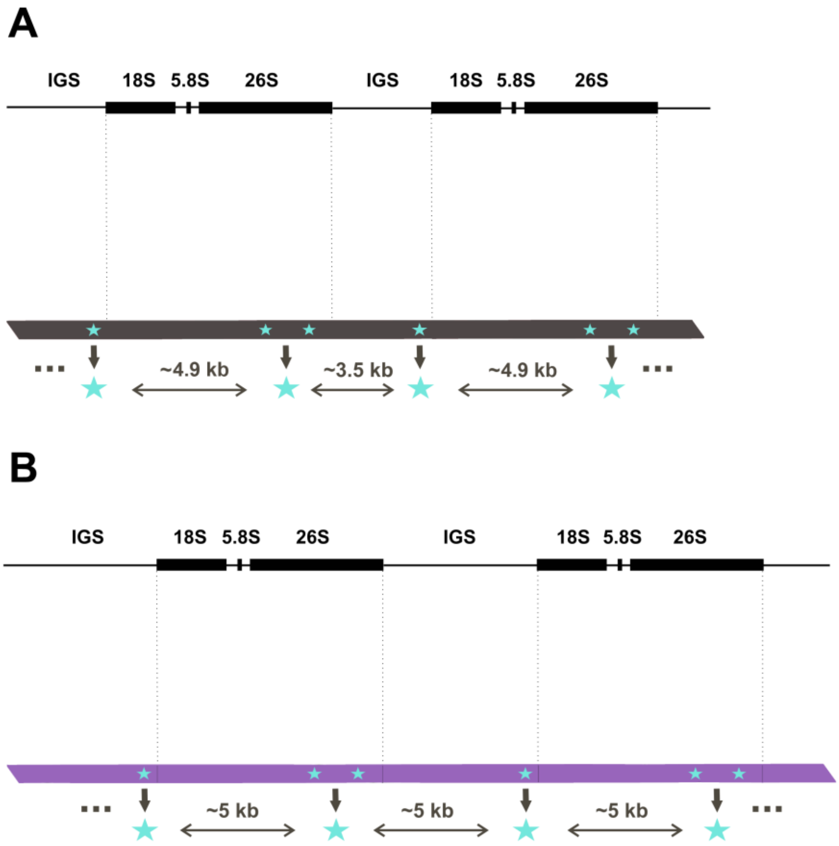

Figure S6. Optical map label pattern of the 45S rDNA array in barley chromosome 1H. (A) Label spacing (big blue stars) predicted *in silico* from the reconstructed sequence of 1H-specific rDNA unit (grey bar). The small stars correspond to recognition sites of Nt.*BspQI* nickase used for labelling. The two nearby sites in the 26S rRNA gene cannot be discriminated and merge into a single signal. (B) Observed label spacing (big blue stars) in 'Morex' OM contig 310 (violet bar). The discrepancy between the prediction and the reality is likely due to incompleteness of the IGS in the reconstructed rDNA unit.

Table 1. Primers for 7DS-specific tandem repeats.

Tandem repeat	Primer name	Primer sequence
**TaeCsTr99**	**Tae7DS99c14-F1**	TTGAGACCTAAAGTTCATATCCACAC
	**Tae7DS99c14-R1**	TATATTTATTCGTACATGTGCTTCCAC
**TaeCsTr111**	**Tae7DS111c48-F**	AAATGGTTAGAAAATCACTTAAATGTC
	**Tae7DS111c48-R**	ATTTGAACTACATCTACATGCAACG
**TaeCsTr163**	**Tae7DS163c25-F**	GGGCAACTAATGGTTTGTAAGC
	**Tae7DS163c25-R**	CCTGAAGACGAGGTAATTTACTGTATC
**TaeCsTr230**	**Tae7DS230c2-F**	AAATTCAAGCCCAGCTAGCAC
	**Tae7DS230c2-R**	AAACTATAGCATTATAGGAGGCAAATG

File S1. FASTA file containing sequences of all tandem repeats.

File S2. FASTA file containing sequences of nanopore reads.

File S3. FASTA file containing sequence of the reconstructed 45S rDNA unit from 1H chromosome of barley cv. Morex.
